# Clinical photon-counting computed tomography in living patients detects intra-plaque haemorrhage and thrombus in carotid plaques

**DOI:** 10.1093/eurheartj/ehaf707

**Published:** 2025-08-29

**Authors:** Annelie Shami, Jiangming Sun, My Celander, Ana Persson, Andreas Edsfeldt, Martin Bech, Marie-Louise Aurumskjöld, Isabel Gonçalves

**Affiliations:** Department of Clinical Sciences Malmö, Lund University, Clinical Research Centre, Malmö, Jan Waldenströms gata 35, CRC 91:12, Malmö 214 28, Sweden; Department of Clinical Sciences Malmö, Lund University, Clinical Research Centre, Malmö, Jan Waldenströms gata 35, CRC 91:12, Malmö 214 28, Sweden; Department for Medical Radiation Physics, Clinical Sciences Lund, Lund University, Lund, Sweden; Department of Clinical Sciences Malmö, Lund University, Clinical Research Centre, Malmö, Jan Waldenströms gata 35, CRC 91:12, Malmö 214 28, Sweden; Department of Clinical Sciences Malmö, Lund University, Clinical Research Centre, Malmö, Jan Waldenströms gata 35, CRC 91:12, Malmö 214 28, Sweden; Department of Cardiology, Skåne University Hospital, Lund University, Carl-Bertil Laurells gata 9, Malmö 214 28, Sweden; Wallenberg Centre for Molecular Medicine, Lund University, Lund, Sweden; Department for Medical Radiation Physics, Clinical Sciences Lund, Lund University, Lund, Sweden; Medical Radiation Physics, Department of Clinical Sciences Malmö, Skåne University Hospital, Lund University, Skåne University Hospital, Malmö, Sweden; Radiation Physics, Department of Hematology, Oncology and Radiation Physics, Skåne University Hospital, Lund, Sweden; Department of Clinical Sciences Malmö, Lund University, Clinical Research Centre, Malmö, Jan Waldenströms gata 35, CRC 91:12, Malmö 214 28, Sweden; Department of Cardiology, Skåne University Hospital, Lund University, Carl-Bertil Laurells gata 9, Malmö 214 28, Sweden

**Keywords:** Atherosclerosis, Plaque, Computed tomography, X-ray, Carotid arteries

## Introduction

Thrombus formation following plaque rupture or erosion is the most common cause of highly fatal cardiovascular events, such as heart attacks and strokes.^1^ Non-invasive evaluation of high-risk plaques through energy-integrated computed tomography has been widely used to estimate calcification and overall stenosis degree; however, additional plaque features, including intra-plaque haemorrhage (IPH), have been proposed as even more relevant for rupture risk assessment.^2^ Photon-counting computed tomography (PCCT), a most recent computed tomography (CT) modality, has the potential for more precise detection through acquisition of high-resolution spectral information with reduced electronic noise.^3^

Specifying the still undetermined possibilities for plaque evaluation by PCCT would add great clinical value through improved diagnostics and risk stratification. This study is an *in vivo* expansion on our previous finding that PCCT enables differentiation of IPH and thrombus, besides calcium, in human carotid plaques.^4^ This is the first study to evaluate detection of high-risk features in carotid plaques using a clinical PCCT protocol in patients.

## Methods

**Figure 1:**
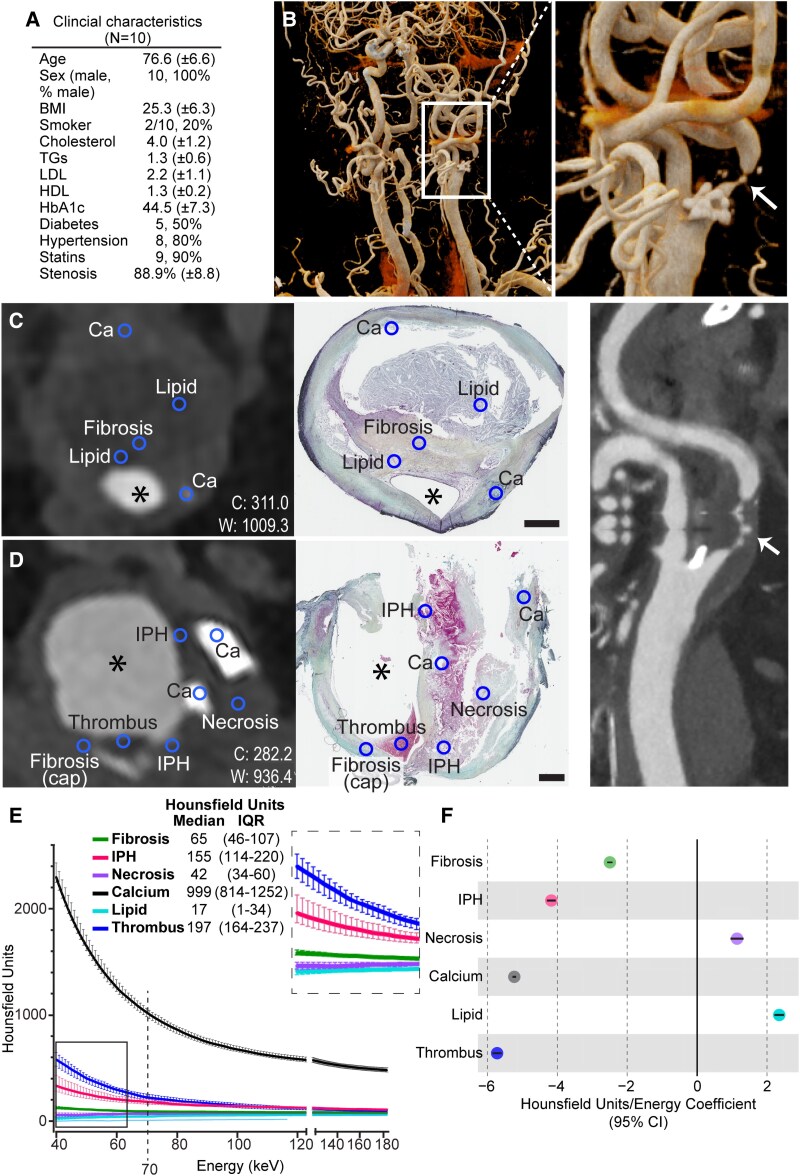
Comparison of the attenuation of distinct atherosclerotic plaque features scanned by photon-counting computed tomography of living patients pre-operatively and the corresponding microscopic images of the plaques obtained by carotid endarterectomy. (*A*) Patient characteristics are shown. Continuous measurements are shown as mean with standard deviation. Categorical variables are shown as absolute numbers (indicating ‘yes’) and percentages (indicating ‘yes’, if not otherwise specified). Body mass index is shown as kg/m^2^. Fasting lipoproteins (cholesterol, triglycerides, LDL, and HDL) are shown as mmol/L, while HbA1c is shown as mmol/mol. *N* = 10, except *n* = 8 for LDL and HDL. High-resolution photon-counting computed tomography angiography overview volume rendering technique images are shown in (*B*) with zoomed region (white box) denoting a high degree of stenosis in the left *carotis interna* (indicated by white arrows). Regions of interest (annotated circles) are shown in representative corresponding images acquired from photon-counting computed tomography scanning (70 keV) and through histochemistry (Russell-Movat’s pentachrome stain) of two different plaques in (*C*) and (*D*). The lumen is denoted by an asterisk, and scale bars represent 1 mm. Measured Hounsfield units (with a 95% confidence interval) are shown for all measured energies (*E*; 40–190 keV, note the segmented *X* axis), along with Hounsfield unit ranges resulting from this dataset at 70 keV, an energy level well within the interval of sufficient statistical power for all the studied plaque feature comparisons [except for intra-plaque haemorrhage (IPH) vs thrombus, where slightly lower power (0.761) is achieved]. Annotated plaque features are compared using the Kruskal–Wallis test followed by Dunn’s *post-hoc* test and Bonferroni correction for multiple comparisons. Hounsfield units measured for calcifications were distinguishable from that of all other features at all energy levels (*P*-values ranging from 2.0 × 10^−123^ to 2.0 × 10^−127^), as was Hounsfield units for intra-plaque haemorrhage (*P* = 2.0 × 10^−20^ to 2.0 × 10^−29^), with the exception of comparison with thrombus. Thrombus Hounsfield units were distinct from all features (excluding intra-plaque haemorrhage) at 40–166 keV (*P* = .049 to 8.0 × 10^−21^), while indistinguishable from fibrosis >167 keV. Besides calcification, intra-plaque haemorrhage, and thrombus, Hounsfield units for fibrosis could be differentiated from lipid core at all energy levels (*P* = 5.0 × 10^−9^ to 1.0 × 10^−15^) and from necrosis Hounsfield units at 40–86 keV (*P* = .047 to .0007), while lipid core and necrosis Hounsfield units were only weakly distinguishable (*P* = .043–.049) from each other at 80–116 and 150–190 keV (except for at 184 keV). In (*F*), differences in coefficients derived through a mixed-effects model were found for all evaluated plaque features (*P* < 9.7 × 10^−7^, shown with 95% confidence interval), as assessed by *t*-test (with Benjamini–Hochberg adjustment for multiple comparisons). *n* (calcium) = 340, *n* (intra-plaque haemorrhage) _=_ 106, *n* (thrombus) = 43; *n* (lipid core) = 107, *n* (fibrosis) = 470, *n* (necrosis) = 62

Matched PCCT and histological imaging of 10 carotid endarterectomy plaques from 10 patients with pre-operative cerebrovascular symptoms (the Carotid Plaque Imaging Project, July to November 2024) were characterized in this cross-sectional, observational, retrospective study (*Figure 1A*). Indications for surgery and symptoms were described previously.^5^ Inclusion criteria were age >18 years, eligibility for carotid endarterectomy and ability to provide informed consent. As a proof-of-concept study, power calculation was not possible.

Photon-counting computed tomography imaging was performed on average 1.7 days (standard deviation 1.6) before endarterectomy with and without an iodine-based contrast agent (omnipaque, 350 mg I/mL) with mirrored scan parameters, using a first-generation clinical dual-source PCCT scanner (NAEOTOM Alpha; Siemens Healthineers GmbH, Forchheim, Germany) in single-source, multi-energy mode (High Resolution Quantum Plus). The scan parameters included a tube potential of 120 kVp, image quality level of 150, and automatic exposure control (CARE Dose4D) enabled. The collimation was 120 × 0.2 mm, with rotation time 0.5 s and pitch 0.85. Images were reconstructed using Qr40 and Bv64 kernels, with a quantum iterative reconstruction (QIR) strength of 4, and using Qr40 and Br84 kernels with QIR 3, for scans with and without contrast, respectively. The reconstructed slice thickness and increment were both 0.4 and 0.2 mm, respectively.

Plaques were formalin-fixed immediately following surgery, paraffin-embedded, and cut cross-sectionally in 3-mm fragments. Histological stains were performed as previously described.^4^ Corresponding plaque locations on histological sections and PCCT slices were matched through the location of the bifurcation, recorded plaque length measurements and topological landmarks. Matching 0.1 mm^2^ regions of interest (ROIs) (*n* = 1128) were manually placed on PCCT (viewed by Syngo.via version VB80D, Siemens Healthineers GmbH) and histological images by a blinded medical physicist. Alignment was manually reviewed and validated by an imaging cardiologist with >20 years of experience interpreting histopathological plaque images and >10 years of experience interpreting CT. The blinded assessment of inter-observer variability gave an overall agreement of 82%. Regions of interest were registered for calcium (*n* = 340; von Kossa), IPH (*n* = 106; glycophorin A), luminal thrombus (*n* = 43; glycophorin A), lipid core (*n* = 107; oxLDL), fibrosis (*n* = 470; Russell-Movat’s pentachrome stain), and necrosis (*n* = 62; plaque regions devoid of cells/tissue).

The Kruskal–Wallis test, followed by Dunn’s *post-hoc* test with Bonferroni correction for multiple comparisons, was used to compare Hounsfield units (HUs) between plaque features. A mixed-effects model was applied to examine the relationship between logarithmically (log) transformed energy and HU for each plaque feature. The obtained coefficients for log-transformed energy, representing HU value changes across energy levels, were compared by *t*-test to assess differences among plaque features (with the Benjamini–Hochberg procedure to control the false discovery rate). An adjusted *P*-value of <.05 was considered statistically significant. Statistical analyses were conducted using R version 4.4.3 (R Core Team 2021, R Foundation for Statistical Computing, Vienna, Austria).

## Results

Photon-counting computed tomography scanning of 10 male patients with pre-operative cerebrovascular symptoms was performed using clinical routine acquisition settings (*Figure 1B*). Regions of interest were placed in corresponding positions on images acquired by PCCT and histologically stained sections denoting macro-calcification, IPH, thrombus, lipid core, fibrosis, and necrosis (*Figure 1C* and *D*), all representing plaque features with previously well-known associations to plaque rupture.^2,6–8^

For each plaque feature category, ROIs were aggregated per recorded keV level (40–190, in 1 keV increments; *Figure 1E*) and medians were compared. Calcification differed from all other annotated plaque features at all energy levels (*P* < .05), as did IPH, except proving indistinguishable vs thrombus. Thrombus was distinguishable from all features at 40–166 keV (except, as expected, from IPH). Moreover, fibrosis could be differentiated from lipid core at all energy levels and from necrosis at 40–86 keV, while lipid core and necrosis were only weakly distinguishable at 79–118 and 150–190 keV.

Finally, plaque features exhibited distinct patterns in the relationships between HU and energy levels, represented by the coefficient for the logarithmic terms from a mixed-effects model (*P* < 9.7 × 10^−7^; *Figure 1F*).

## Discussion

This is the first PCCT study to characterize human carotid plaque features in living patients, identifying differentiating spectral patterns between calcium, IPH, thrombus, fibrosis, necrotic, and lipid core, while using standard contrast and PCCT protocols to explore the richness of the various energy levels, fully aligning with clinical practice.

While current clinical assessment still relies mostly on the degree of stenosis, the risk of cardiovascular events is considered to be at least as dependent on other plaque features such as IPH, lipid-richness, or necrotic core.^2,6–8^ Objective quantification of such plaque elements in the carotids has been described using classical CT angiography, e.g. by Buckler *et al*.,^9^ but similar reports are scarce for comparable PCCT assessment^10^ and so far limited to *ex vivo* observations.^3^

Study limitations include patients with carotid (and not coronary) disease of the same sex and ethnicity, with similarly high stenosis degrees. Generalizability is limited due to the overall study design, a limited number of subjects (though with a high number of evaluated ROIs), and lack of comparative accuracy with other imaging modalities (e.g. standard CT, magnetic resonance) or clinical characteristics.

In conclusion, clinical PCCT scans from living patients allow, beyond calcium, the detection of IPH and thrombus, well-known components relevant to plaque rupture risk. This represents an important advance in non-invasive plaque imaging with the potential to improve diagnostics, risk stratification, and patient monitoring, ultimately reducing cardiovascular mortality and disability.

## Declarations

### Data Availability

Sharing of pseudonymized participant data is subject to limitations specific to the ethical permit and general data protection regulation [GDPR (EU) 2016/679].

### Pre-registered Clinical Trial Number

The pre-registered clinical trial number is NCT05821894 (ClinicalTrials.gov).
